# Investigating the antimicrobial and anti-inflammatory effects of *Lactobacillus* and *Bifidobacterium* spp. on cariogenic and periodontitis pathogens

**DOI:** 10.3389/fmicb.2024.1383959

**Published:** 2024-05-30

**Authors:** Marzie Mahdizade Ari, Shiva Mirkalantari, Davood Darban-Sarokhalil, Atieh Darbandi, Shabnam Razavi, Malihe Talebi

**Affiliations:** ^1^Department of Microbiology, School of Medicine, Iran University of Medical Sciences, Tehran, Iran; ^2^Microbial Biotechnology Research Center, Iran University of Medical Sciences, Tehran, Iran

**Keywords:** probiotics, *Lactobacillus*, *Bifidobacterium*, oral health, dental caries, periodontitis, inflammation

## Abstract

**Background:**

The use of probiotics is emerging as an innovative approach to managing oral health issues and mediating the immune system. The current study assessed the *in vitro* impacts of non-orally isolated probiotics on periodontitis and tooth decay pathogens.

**Methods:**

Briefly, the persistence of probiotics in exposure to oral cavity enzymes, hydrogen peroxide, and saliva samples was examined. It was also investigated the biofilm formation and aggregation ability of probiotics, the adherence of probiotics in human gingival fibroblast cell (HGFC) lines and molar teeth samples, and the potential of probiotics to co-aggregate with oral pathogens. Additionally, the current study evaluated the effects of live probiotics on virulence gene expression, biofilm production of main oral pathogens, and changes in inflammation markers.

**Results:**

The probiotics remained alive when exposed to enzymes in the oral cavity, hydrogen peroxide, and saliva at baseline, 1, 3, and 5 h after incubation at 37°C (*p-*value <0.05). Probiotics demonstrated to produce biofilm and aggregation, as well as adherence to HGFCs and maxillary molars (*p-*value >0.05). They showed significant co-aggregation with oral pathogens, which were recorded as 65.57% for *B. bifidum* 1001 with *S. mutans*, 50.06% for *B. bifidum* 1005 with *P. gingivalis*, 35.6% for *L. plantarum* 156 with *F. nucleatum*, and 18.7% for *B. longum* 1044 with *A. actinomycetemcomitans* after 8 h of incubation. A balance between pro-inflammatory and anti-inflammatory cytokines, along with inhibition of biofilm formation and changes in virulence gene transcripts, were observed. However, most of these changes were not statistically significant (*p-*value >0.05).

**Conclusion:**

This study demonstrated the direct link between adhesiveness, aggregation, and biofilm formation with probiotic antibacterial activity. In addition to the careful selection of suitable probiotic strains, the concentration and origin of probiotic isolates should be considered.

## 1 Introduction

Probiotics, when administered in adequate quantities, are live microorganisms that significantly impact human health (Saraf et al., 2010). These beneficial bacteria need to withstand the harsh environment of the gastrointestinal tract. Probiotics directly fight pathogens by producing antibacterial compounds such as bacteriocins, and various metabolites like organic acids (Ibarburu et al., 2015). Probiotics are widely utilized in food, pharmaceutical, chemical industries as well as cosmetic. Several types of microorganisms are introduced as probiotics, such as *Lactobacillus acidophilus* (*L. acidophilus*), *Lactiplantibacillus plantarum* (*L. plantarum*), *Lacticaseibacillus casei* (*L. casei*), *Lactobacillus delbrueckii* (*L. delbrueckii*), *Lacticaseibacillus rhamnosus* (*L. rhamnosus*), *Limosilactobacillus fermentum* (*L. fermentum*), *Limosilactobacillus reuteri* (*L. reuteri*), and *Lacticaseibacillus paracasei* (*L. paracasei*), *Bifidobacterium bifidum* (*B. bifidum*), *Bifidobacterium breve* (*B. breve*), *Bifidobacterium longum* (*B. longum*), *Bifidobacterium lactis* (*B. lactis*), and *Bifidobacterium animalis* (*B. animalis*). *Streptococcus* spp.; *Enterococcus*; *Saccharomyces*; *Pediococcus;* and *Leuconostoc* are also recognized for their probiotic properties (Padmavathi et al., 2018). Probiotics have been found to maintain the stability and diversity of oral biofilms by interacting with the oral microbial population, potentially alleviating symptoms of metabolic disorders, cancer, allergic reactions, and autoimmune disorders (Menon, 2016). The precise mechanism of action of probiotics in the oral cavity is not fully understood, and it is still under investigation. Studies suggested that the primary function of probiotics involves competing for attachment sites, producing of metabolites against pathogens, and regulating immune responses. In particular, *L. casei* strain *Shirota* and *L. reuteri* have been shown to effectively hinder the growth and biofilm formation of *Streptococcus mutans* (*S. mutans*) and *Porphyromonas gingivalis* (*P. gingivalis*) (Widyarman et al., 2019). Moreover, the levels of pro-inflammatory cytokines like interleukin-1 beta (IL-1β), tumor necrosis factor alpha (TNF-α), and interleukin-8 (IL-8) decreased in the gingival crevicular fluid (GCF) following chewing gum containing *L. reuteri* (Menon, 2016). Periodontitis is a severe infectious disease that impacts the tooth-supporting tissues and is characterized by bleeding and redness of the gums. Apart from the enrollment of host factors in periodontitis, the main reason behind periodontitis is attributed to a combination of virulence factors present in pathogens responsible for colonization, tissue destruction, and biofilm formation. Among all the factors mentioned earlier, this stands out as the primary and crucial element in the colonization. *Aggregatibacter actinomycetemcomitans* (*A. actinomycetemcomitans*), *P. gingivalis*, and *Fusobacterium nucleatum* (*F. nucleatum*) are the etiological causative agents of periodontitis (Sujeetha et al., 2019). Tooth decay, another frequent oral health issue, occurs when a sticky mass known as dental plaque or biofilm develops on the surface of the teeth. During this procedure, certain species of *Streptococcus*, including *S. mutans* and *S. sobrinus*, play a role in creating substances like glucan and fructan (Lee and Kim, 2014), and increasing biofilm-producing proteins to enhance bacterial biofilm formation ability (Matsumoto-Nakano, 2018). Periodontal disease occurs when the balance of microorganisms in the oral cavity is disturbed by the excessive growth of harmful periopathogens and the formation of biofilm. This process ultimately causes the development of periodontal pockets, damage to the surrounding tissues, and deterioration of the bone supporting the teeth (Cugini et al., 2021). It has been suggested that probiotics have the ability to treat and prevent periodontitis and dental caries. To select the most effective probiotic for combating oral pathogens, we conducted an *in vitro* study to explore the effects of probiotics isolated from milk and feces on the expression of minor fimbriae (*Mfa1*) and arginine-gingipain (*Rgp*) genes in *P. gingivalis*, rough-colony protein A (*RcpA*) in *A. actinomycetemcomitans*, glucosyltransferase B (*GtfB*) in *S. mutans*, and fibroblast activation protein-2 (*Fap2*) in *F. nucleatum;* the formation of biofilms (microbial plaque) by oral pathogens; as well as the inflammation in the oral cavity, which characterized by inflammatory cytokines like IL-8, and anti-inflammatory cytokines like IL-10. Before that, it is important to address the effectiveness and viability of probiotics in the rough condition of the oral cavity.

## 2 Materials and methods

### 2.1 Study design

The effectiveness of probiotics in the oral cavity was assessed when exposed to various enzymes, hydrogen peroxide, and saliva. The ability of probiotics to attach to human gingival fibroblast cells (HGFCs) and maxillary teeth was also analyzed. In the following section, the effect of each strain of *Bifidobacterium* and *Lactobacillus* spp., a combination of five *Lactobacillus* strains, a combination of five *Bifidobacterium* strains, and a combination of the most effective *Lactobacillus* and *Bifidobacterium* strains were analyzed on the expression of the main virulence genes in *A. actinomycetemcomitans*, *P. gingivalis*, *F. nucleatum*, and *S. mutans*. Analysis of the gene expression of IL-8 and IL-10 cytokines was also conducted on the supernatant of HGFC. Except for the competition and biofilm formation assays, which were carried out in duplicate, all other experiments were performed in triplicate.

### 2.2 Bacterial strains and growth conditions

Five *Lactobacillus* strains were isolated from the stool and five *Bifidobacterium* strains were taken from breast milk and infant feces. These strains were chosen for investigation in the current study, as outlined in Table 1. The study included *Lactiplantibacillus plantarum* (*L. plantarum* 42 and *L. plantarum* 156), *Levilactobacillus brevis* (*L. brevis* 205), *Limosilactobacillus reuteri* (*L. reuteri* 100), and *Lacticaseibacillus rhamnosus* (*L. rhamnosus* 195) (Rohani et al., 2015). Additionally, *Bifidobacterium bifidum* (*Bifidobacterium bifidum* 1001 and *Bifidobacterium bifidum* 1005), *Bifidobacterium breve* (*Bifidobacterium breve* 1015 and *Bifidobacterium breve* 1063), and *Bifidobacterium longum* (*Bifidobacterium longum* 1044) were also studied (Eshaghi et al., 2017). The current study involved the use of four pathogens, including *A. actinomycetemcomitans* JP2 genotype strain HK1651 and *S. mutans* purchased from the microbial collection bank of Shahid Beheshti University of Medical Sciences, faculty of dentistry, as well as *P. gingivalis* ATCC 33277 and *F. nucleatum* ATCC 27725 from Tehran University of Medical Sciences (kindly donated by Dr. Douraghi).

**Table 1:** Characteristics of the probiotics which used in present study.

Strains	pH = 2	Bile salt (0.4%)	Antimicrobial activity	Safety hemolysis
*Lactiplantibacillus plantarum* (42)	R	R	+++++	Safe
*Lacticaseibacillus rhamnosus* (195)	R	R	+++++	Safe
*Levilactobacillus brevis* (205)	R	R	++	Safe
*Lactiplantibacillus plantarum* (156)	R	R	+++++	Safe
*Limosilactobacillus reuteri* (100)	R	R	++	Safe
*Bifidobacterium bifidum* (1001)	R	R	++	Safe
*Bifidobacterium bifidum* (1005)	R	R	++	Safe
*Bifidobacterium breve* (1015)	R	R	+++++	Safe
*Bifidobacterium breve* (1063)	R	R	++	Safe
*Bifidobacterium longum* (1044)	R	R	++	Safe

Probiotics were grown on Man-Rogosa-Sharpe (MRS) agar (Merck, Darmstadt, Germany) and incubated at 37°C for 48–72 h under microaerophilic conditions. *A. actinomycetemcomitans* and *P. gingivalis* were cultivated on brain heart infusion (BHI) agar (Merck, Darmstadt, Germany) supplemented with 0.5% defibrinated sheep blood and incubated at 37°C for 48–72 h under anaerobic conditions. *F. nucleatum* was grown on Brucella agar based medium (Condalab, Spain) supplemented with 5% horse serum (Baharafshan, Iran) and 10% defibrinated sheep blood (Baharafshan, Iran) and incubated at 37°C for 48–72 h in anaerobic conditions. *S. mutans* was cultured on blood agar (Merck, Darmstadt, Germany) and incubated at 37°C for 24 h under aerobic conditions. In all experiments, to achieve an optical density (OD) of 0.08–0.13 equivalent to 1.5 × 10^8^ colony forming units (CFUs)/mL, bacterial suspensions were first prepared by transferring fresh colonies of probiotics and pathogens grown on agar plates to MRS and BHI broth, respectively, and then incubated at 37°C under microaerophilic conditions.

### 2.3 Probiotics viability assays in the oral cavity

#### 2.3.1 Effect of hydrogen peroxide on the survival of probiotics

Suspensions of *Lactobacillus* and *Bifidobacterium* strains were prepared at a concentration of 1.5 × 10^8^ CFU/mL, exposed to 0.4 mM H_2_O_2_ (30% w/v) (Mojallali, Iran), and then incubated at 37°C for 18 h in the presence of 5% CO_2_ under microaerophilic conditions. Survival of probiotics was evaluated at different time intervals at baseline, 1, 3, and 5 h of incubation using a colony count assay. Following incubation, each treatment was subjected to serial dilution and spot culture on MRS agar to determine the survived strains, and then incubated at 37°C for 24 h. After 24 h, colonies growing on MRS agar that belonged to *Lactobacillus* or *Bifidobacterium* spp. were counted (Sun et al., 2013).

#### 2.3.2 Effect of protease, lysozyme, lipase, and *α*-amylase on the survival of probiotics

Probiotics at a concentration of 1.5 × 10^8^ CFU/mL were treated with PBS (phosphate-buffered saline) (Bio-IDEA, Iran) containing α-amylase (220 IU/mg), lipase (700 IU/mg), lysozyme (22 IU/mg), and proteinase K (1 mg/mL) (Mojallali, Iran) (Yang et al., 2021). NaOH was introduced to enhance the effectiveness of *α*-amylase and lipase by adjusting the pH level to 6.5. A colony count assay was performed to evaluate the survival rate of the treated probiotics compared to the control group (probiotics without enzymes) at baseline, 1, 3, and 5 h after incubation at 37°C.

### 2.4 Growth assay of probiotics in saliva

#### 2.4.1 Saliva preparation

Briefly, 1.5 h after eating, drinking, or tooth brushing, 3 mL of unstimulated saliva from healthy volunteers was collected in sterile tubes (MAXWELL, China), and immediately placed on an ice pack. The collected saliva sample was centrifuged (Beckman, United States) at 10,000 rpm and 4°C for 10 min (min) to obtain a homogenous sample. Subsequently, 1 mL of the supernatant was carefully transferred into an Eppendorf tube. Then, 10 μL of supernatant was placed on blood agar and kept for incubation in both aerobic and anaerobic conditions. In order to obtain germ-free saliva and prevent any interference with the probiotic treatment process, the supernatant was pasteurized by heating at 65°C for 30 min. Following centrifugation, the supernatant was transferred to a new tube, and kept at −70°C. Before that, 10 μL of pasteurized supernatant was cultured on blood agar, and incubated in aerobic and anaerobic conditions to assess for any potential contamination.

#### 2.4.2 Treatment of probiotics with saliva

A fresh overnight culture of probiotics was prepared at 1.5 × 10^8^ CFU/mL. Then, 200 μL of probiotic suspension was mixed with 1.8 mL of pasteurized saliva and incubated at 37°C for 24 h. Serial dilution was carried out before and after the incubation period. For the control group, the same procedures were followed, while 0.9% NaCl was used instead of saliva. All experiments were repeated twice.

### 2.5 Adhesion test of probiotics to maxillary molar

Intact maxillary molars were provided by healthy women who have been replaced with gold teeth for cosmetic reasons. In order to investigate the adherence ability of probiotics to maxillary molars, suspensions of probiotics at 1.5 × 10^8^ CFU/mL were prepared. Then, 1.5 mL of the prepared suspension was placed into a 2-mL sterile Eppendorf tube containing a sterilized maxillary molar sample, and incubated at 37°C for 48 h under microaerophilic conditions. After incubation, the maxillary molar was rinsed three times with either normal saline or PBS to eliminate unattached bacteria. The maxillary molar was gently washed with PBS and transferred into a 2-mL sterile Eppendorf tube containing 1.5 mL of PBS. It could be either sonicated for 15 s at a frequency of 30 kHz and an output power of 7 W or centrifuged at 5,000 rpm and 4°C for 3–5 min to remove unbound and residual periopathogens (Pourhajibagher et al., 2020). Finally, 10 μL of Eppendorf content containing maxillary molar was placed on MRS agar. After incubation at 37°C for 72 h under microaerophilic conditions, the CFU/mL of the probiotic strain adhered to the maxillary molar was calculated based on the method described by Miles et al. (1938).

### 2.6 Biofilm formation ability of probiotics

The biofilm formation ability of probiotics was evaluated according to Mirzaei et al. (2022) using a microtiter plate assay. Briefly, the suspension of fresh probiotics was cultured in MRS broth supplemented with 2.5% glucose and incubated at 37°C for 12–36 h with 5% CO_2_. Then standard suspensions (1.5 × 10^8^ CFU/mL) of probiotics were prepared, and 200 μL of suspension containing probiotic-MRS broth was added to a 96-well microplate (NEST, China) and incubated in an incubator with 5% CO_2_ at 37°C for 36 h. After incubation, the suspension was gently removed from the well, and washed three times with PBS and air-dried for 30 min. Then, 200 μL of methanol (Mojallali, Iran) was added to each well to stabilize the biofilm. After 15 min, the solution was aspirated, and the plate was air-dried at room temperature. The wells were stained with 200 μL of crystal violet (0.05%) for 5 min, and the solution was aspirated, and the wells were washed three times with PBS and left to dry naturally at room temperature for 30 min. Finally, 200 μL of ethanol (95%) (Mojallali, Iran) was added to each well and incubated at 37°C for 30 min in a shaker incubator (IKA, Germany). The content of each well was transferred to the corresponding well in another microplate, and the absorbance was measured at 492 nm using a microplate reader (BioTek, United States). All experiments were performed in triplicate. The classification of probiotic isolates based on their optical absorbance (OD) is showed in Table 2.

**Table 2:** Optical absorbance of the strain (OD) and optical absorbance of the well (ODc).

The optical absorption	Binding results
No attachment	OD < ODc
Weak attachment	ODc < OD < 2OD
Medium attachment	2ODc < OD < 4ODc
Strong attachment	OD4 < ODc

### 2.7 Aggregation and co-aggregation ability of probiotics

Aggregation assay was performed according to Del Re et al. (2000). Certain probiotics with the ability to form aggregates, can effectively inhibit the formation of dental plaque by biofilm-producing bacteria. Briefly, probiotic isolates were incubated overnight in MRS broth medium in an incubator with 5% CO_2_ at 37°C. After washing the cells with phosphate buffered saline (PBS), the probiotic suspension was prepared with PBS to achieve a concentration of 1.5 × 10^8^ CFU/mL. The ability of the current probiotics to induce aggregation was investigated by measuring the decrease in optical absorbance of bacterial suspensions at 600 nm due to aggregation and diffusion. The aggregation capacity (AC) was determined using the following formula, where ODt and OD0 are the optical absorbance at the end (8 h) and initial times, respectively (Bosch et al., 2012).


$$\mathrm{AC}=\frac{1-\left(ODt/OD0\right)}{100}$$

The methods employed for co-aggregation and aggregation assays were identical. Briefly, probiotic isolates were incubated overnight in MRS broth in an incubator with 5% CO_2_ at 37°C. After washing the cells with phosphate buffered saline (PBS), the probiotic suspension was prepared with PBS to achieve a concentration of 1.5 × 10^8^ CFU/mL. The capability of the probiotics to co-aggregate with pathogens was investigated by combining equal quantities of probiotics and pathogens. After 8 h, the turbidity of the mixture was examined and compared to that of individual suspensions of each pathogen and each probiotic as the control groups (MacDonald et al., 2021). The co-aggregation capacity was reported as the percentage reduction in optical absorbance and calculated according to Handley et al. (1987) using the following formula: Where *X* and *y* indicate each of the two strains in the control tubes, (*x* + *y*) represents the mixture, and A represents absorbance at a wavelength of 600 nm.


$$Co-\mathrm{aggregation}=\frac{\frac{\mathrm{AX}+AY}{2}-A\left(X+Y\right)}{\left(\mathrm{AX}+AY\right)/2}\times 100$$

### 2.8 Human gingival fibroblast cell (HGFC) culture

The HGFC cells with the NCBI code of C165 were obtained from the Cell Bank Department of the Pasteur Institute of Iran. The HGFC were grown in DMEM (Dulbecco’s Modified Eagle Medium) (Biosera, France) supplemented with 15% heated-inactivated fetal bovine serum (FBS) (Biosera, France) and 2% glutamine (Bio-IDEA, Iran). Then, the cells were incubated in a 5% CO_2_ incubator at 37°C. HGF cells were seeded in 6-and 12-well plates (SPL, Korea) in order to conduct adhesion and competition assays, respectively.

#### 2.8.1 The MTT cell viability assay

The MTT (3-(4,5-dimethylthiazol-2-yl)-2,5-diphenyltetrazolium bromide, a tetrazole) assay was used to determine the potential inhibitory effects of probiotics on HGFCs. MTT determines the survival rate of cells based on cell metabolic activity after treatment with drugs or agents. A living cell has a mitochondrial dehydrogenase enzyme which converts tetrazolium into formazan, and the color of cell changes to purple following the cleavage of tetrazolium to into formazan. Finally, color intensity is determined based on optical absorbance. Briefly, the cells were seeded at a concentration of 2.5 × 10^4^ cells per well in 96 well plates. At a confluency of 70–90%, cells were treated with 250 μL of probiotics and incubated in an incubator with 5% CO2 at 37°C for 1, 3, and 24 h. To reduce MTT into formazan, 100 μL of MTT (Sigma, United States) was added to each well containing cells and probiotics and incubated in an incubator with 5% CO2 at 37°C. After incubation, 100 μL of DMSO lysing solution was added to each well to lyse the insoluble formazan crystals and release the formazan. The contents of the wells were transferred to a sterile 96-well microtiter plate, and the optical absorbance (optical density, OD) was read at 490 nm using a microplate reader (BioTek, United States). To determine the percentage survival rate of HGFC, the OD of the treated, untreated, and blank wells was measured using the following formula:

(AB: absorbance of blank well, AC: absorbance of control, and AT: absorbance of treated well)


$$\mathrm{Cell}\;\mathrm{Survival}\;\mathrm{Rate}=\frac{AT-\mathrm{AB}}{\mathrm{AC}-\mathrm{AB}}\times 100$$

#### 2.8.2 Adhesion assay of probiotics to HGFCs

The adhesion potential of bacteria to surfaces is studied using qualitative and quantitative approaches. In the qualitative method, the number of attached bacteria is determined using the gram staining technique with a light microscope, and the findings are reported as a percentage. In the quantitative method, the bacteria attached to the well are transferred to a plate containing agar medium. Following the incubation, the number of bacteria in the plate is determined using serial dilution and colony count methods. This study examined the binding ability of probiotics to HGFCs through a qualitative adhesion assay. Briefly, 6-well plates with 70–90% confluency of HGFCs were prepared. After 24 h of incubation, the plate wells were washed three times with PBS, and then 2 mL of fresh high-glucose DMEM medium enriched with 10% FBS was added to each well and incubated at 37°C for 30 min in the presence of 5% CO_2_. A probiotic suspension at a concentration of 1.5 × 10^8^ CFU/mL was prepared using DMEM and transferred to the wells containing 2 mL of HGFCs. The experiment was performed in triplicate, and HGFCs that were not exposed to probiotics were used as a negative control. After 120 min of incubation at 37°C with 5% CO_2_, the wells were slowly aspirated and washed three times with PBS. Then, the gram staining method was performed, and adherent probiotics were counted with a light microscope (X100) in 20 microscopic fields (Zhang and Duan, 2022). Following counting visible probiotics on HGFC surfaces, the adhesion index was calculated by the formula “number of adherent bacteria/number of cells × 100%,” probiotic strains were classified (D'Alessandro et al., 2021).

#### 2.8.3 Gingival fibroblast challenge with probiotics and oral pathogens

In this research, a competition assay was conducted to assess the ability of *Lactobacillus* and *Bifidobacterium* to reduce the virulence of *F. nucleatum*, *P. gingivalis*, *A. actinomycetemcomitans*, and *S. mutans*. To carry out this experiment, 12-well plates containing HGFCs with a confluency of 70–90% were prepared. The wells were rinsed with PBS three times, and, 2 mL of fresh DMEM medium with 10% FBS was added to each well. The plates were then placed in an incubator at 37°C with 5% CO_2_ for 30 min. Bacterial suspensions with a concentration of 1.5 × 10^8^ CFU/mL were prepared from fresh cultures of *Lactobacillus*, *Bifidobacterium*, and pathogens using PBS or DMEM, and introduced to the wells containing 2 mL of HGF cells. Wells with and without bacteria were considered as positive and negative controls, respectively. All analyses were performed in duplicate.

The intervention groups were divided into two main groups; including the treatment and prevention groups. To prepare the treatment group, HGFCs were first exposed to the pathogen, then probiotics were added separately. Conversely, in the prevention group, HGFCs were first treated with probiotics before the pathogen was introduced. Briefly, 200 μL of the probiotics at 1.5 × 10^8^ CFU/mL were added to the wells containing HGFCs and incubated for 60 min, then 200 μL of each pathogen (*F. nucleatum*, *P. gingivalis*, *A. actinomycetemcomitans*, and *S. mutans*) in equal volume was added separately and incubated anaerobically for 60 min at 37°C to evaluate changes in virulence gene expression of pathogens in the presence of probiotics (Haukioja et al., 2008). Similar to the prevention group, 200 μL of the pathogens at a concentration of 1.5 × 108 CFU/mL were added to the wells containing HGFCs and allowed to bind for 60 min of anaerobically incubated at 37°C, then 200 μL of each probiotic with the same concentration was added and incubated for another 60 min (Haukioja et al., 2008).

#### 2.8.4 Evaluating the effect of probiotics on the virulence gene expression of oral pathogens by qRT-PCR

Quantitative real-time polymerase chain reaction (qRT-PCR) was performed to investigate the expression of *Mfa1* and *Rgp* genes in *P. gingivalis*, *RcpA* in *A. actinomycetemcomitans*, *Fap2* in *F. nucleatum*, and *GtfB* in *S. mutans* following exposure to single probiotics, a cocktail of five *Lactobacillus* species, a cocktail of five *Bifidobacterium* species, and cocktail of *Lactobacillus* plus *Bifidobacterium*. REX solution (YTA, Iran) was used to extract bacterial RNA from HGFCs according to the manufacturer’s instructions. Following the extraction of RNA, the purity and quality of the RNA were assessed using a NanoDrop spectrophotometer (Beckman, United States) and agarose gel electrophoresis (Fanavaranakhtarian, Iran), respectively. Genomic DNA was eliminated by RNase free DNase I treatment (Thermo Fisher Scientific, United States), and cDNA was synthesized using cDNA Synthesis kit (Favorgen Biotech, Austria). qRT-PCR was performed using SYBR Green qPCR Master Mix (YTA, Iran) in a final volume of 20 μL. The primers used in this study, were designed and verified by NCBI primer BLAST (Table 3). qRT-PCR thermal cycling conditions were specific for each gene but usually considered as: 95°C for 3 min (initial denaturation), followed by 40 cycles of 95°C for 15 s (denaturation), 54°C for 20 s (annealing), and 72°C for 30 s (extension) conducted on a real-time PCR cycler (Rotor-Gene, Germany). Gene expression levels were calculated according to the 2^-ΔΔCT^ method.

**Table 3:** Primers used in the present study.

Gene name	Primer sequence (5′–3′)	Product size (bp)
IL-10	F: GCCTAACATGCTTCGAGATC	151
	R: TGATGTCTGGGTCTTGGTTC	
IL-8	F: ATGACTTCCAGCTGGCCGTGGCT	292
	R: TCTCAGCCCTCTTCAAAAACTTCTC	
GAPDH	F: GTCTCCTCTGACTTCAACAGCG	131
	R: ACCACCCTGTTGCTGTAGCCAA	
Mfa1	F: CAGATGGGTTGTTGCTCA	150
	R: ATAGAAAGTGCTGCTGGTAG	
RgpA	F: CCGAGCACGAAAACCAA	150
	R: GGGGCATCGCTGACTG	
rcpA	F: ATCCACCTCCGAAACCGAAG	151
	R: TGGGCATTAACTGGAGCCAC	
Fap2	F: GGGGAAATAGGTCGTTCTGC	101
	R: CCAACCCCAACACTTTCATC	
GtfB	F: TGTTGTTACTGCTAATGAAGAA	130
	R: GCTACTGATTGTCGTTACTG	
16srRNA*S. mutans*	F: GTGAAATCCCCGGGCTTAAC	217	R: ACCGTTTACAGCGTGGACTA
16srRNA*P. gingivalis*	F: ACGTCATCCCCACCTTCCTC		197	R: TGTAGATGACTGATGGTGAAAACC
16srRNA*A. actinomycetemcomitans*	F: TTCCGATTAACGCTCGCAC	63		R: AAGCACCGGCTAACTCCGT
16srRNA*F. nucleatum*	F: GGTTAAGTCCCGCAACGA		270	R: CATCCCCACCTTCCTCCTAC

#### 2.8.5 Evaluating the effect of probiotics on IL-8 and IL-10 cytokine changes by qRT-PCR

The HGFC were cultured in DMEM supplemented with 10% FBS (without antibiotics) and seeded in 24-well plates after reaching 70–90% confluency. To induce inflammatory and anti-inflammatory cytokines in the HGF cell line, HGFC were pre-treated with *P. gingivalis* at a MOI of 25:1 and an incubated for 8 h. The probiotics were then added to the pre-treated wells at an equal MOI and incubated in incubator with 5% CO_2_ at 37°C. The wells were washed with PBS to remove any cells that were not bound (MacDonald et al., 2021). The collected supernatants were centrifuged at 2,000 rpm for 5 min. Then, the resulting supernatant was used for the analysis of cytokines. Changes in IL-8 and IL-10 gene expression in HGFC supernatant were evaluated using qRT-PCR. The RNA of the infected cells was extracted using REX solution (YTA, Iran), and cDNA synthesis was done by a cDNA synthesis kit (Favorgen Biotech, Austria). Gene expression levels were calculated according to the 2^-ΔΔCT^ method.

ΔΔCT = ΔCT test sample – ΔCT calibrator sample = 2^-ΔΔCT^

### 2.9 Effect of probiotics on biofilm formation (dental plaque) of oral pathogens

*F. nucleatum*, *P. gingivalis*, *A. actinomycetemcomitans*, and *S. mutans*, along with probiotic strains, were prepared in a solution containing 1.5 × 10^8^ CFU/mL. The equal volumes (200 μL) of the probiotic and the pathogen were cultured in TSB (tryptic soy broth) (Merck, Darmstadt, Germany) with 1% sucrose, then transferred to a 96-well plate and incubated in an incubator with 5% CO_2_ at 37°C. The control group was considered for each pathogen and probiotic. Following the incubation, the suspension was carefully removed from the wells, and the wells were rinsed three times with PBS, before being left to dry for 30 min. Then, 200 μL of methanol was added to each well to stabilize the biofilm. After 15 min, the solution was aspirated, and the plate was air-dried at room temperature. The wells were stained with 200 μL of crystal violet (0.05%) and incubate for 5 min. Then, the solution was aspirated, and the wells were washed three times with PBS and left to air dry for 30 min at room temperature. Finally, 200 μL of ethanol (95%) was added to each well, and left to incubated at 37°C for 30 min in a shaker incubator. The content of each well was transferred to its equivalent well in the same microplate, and the absorbance was measured at 570 nm using a microplate reader.

### 2.10 Statistical analysis

GraphPad software version 9 was used for statistical analysis of the data. Paired-sample *t*-test was used to show significant differences between the groups receiving probiotics and the control group. Dunnet’s two-sided *t*-test was used to investigate the effect of probiotics on gene expression and biofilm formation of periodontal pathogens in the groups receiving probiotics either as prevention or as treatment, which allows multiple comparisons. The statistical significance level of the data was considered to be less than 0.05 (<0.05).

## 3 Results

### 3.1 Survival rate of probiotics in the oral cavity

#### 3.1.1 Hydrogen peroxide

**Figure 1:**
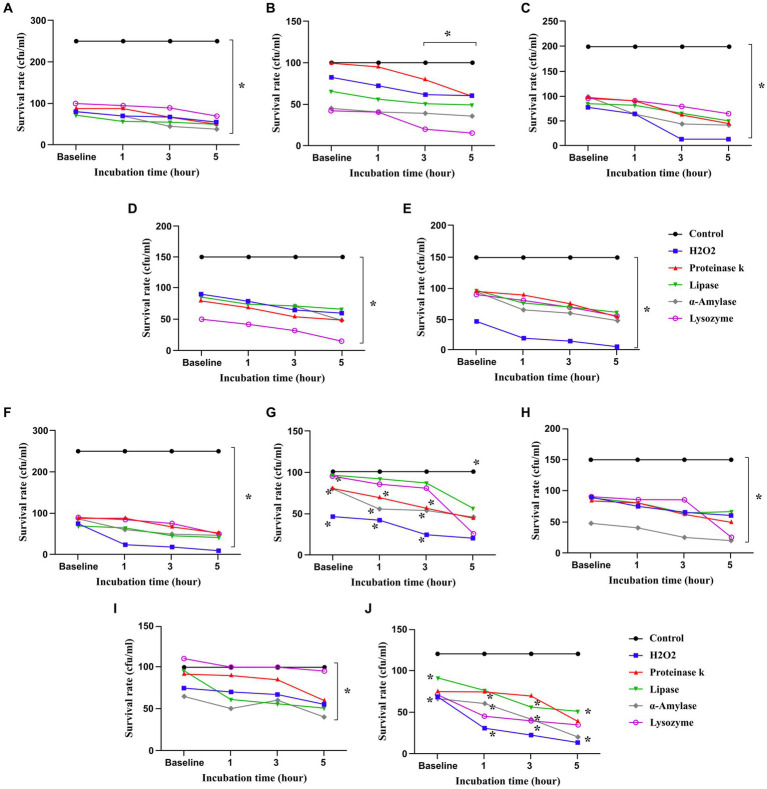
Probiotics survived in presence of hydrogen peroxide (H_2_O_2_), proteinase K, lipase, *α*-amylase and lysozyme. All data describe as mean ± SEM. ^*^p < 0.0001. **(A)** *L. plantarum* 42, **(B)** *L. reuteri* 100, **(C)** *L. rhamnosus* 195, **(D)** *L. brevis* 205, **(E)** *L. plantarum* 156, **(F)** *B. bifidum* 1001, **(G)** *B. bifidum* 1005, **(H)** *B. breve* 1015, **(I)** *B. breve* 1063, **(J)** *B. longum* 1044.

*Lactobacillus* and *Bifidobacterium* strains remained alive at baseline and after being exposed to 0.4 mM H_2_O_2_ for 1, 3, and 5 h (Figure 1). However, *L. reuteri* 100, *L. brevis* 205, *B. breve* 1,015, and *B. breve* 1,063 were more resistant to H_2_O_2_, all strains survived notably up to 5 h after exposure to H_2_O_2_.

#### 3.1.2 Lysozyme, proteinase K, lipase, and *α*-amylase

According to the results shown in Figure 1, *L. rhamnosus* 195, *L. plantarum* 156, *B. bifidum* 1005, *B. breve* 1015, *B. longum* 1044, and *B. breve* 1063 showed great tolerance among other strains when exposed to lysozyme (22 IU/mg). The findings demonstrated a consistent outcome when using proteinase-treated probiotics, suggesting the durability of all strains following exposure for 1, 3, and 5 h. Indeed, *L. reuteri* 100, *L. plantarum* 156, *B. bifidum* 1005, *B. breve* 1015, *B. breve* 1063, and *B. longum* 1044 exhibited the greatest resistance to proteinase. In addition, after being exposed to α-amylase (220 IU/mg), all the probiotic strains, particularly *L. reuteri* 100, *L. rhamnosus* 195, *L. brevis* 205, *L. plantarum* 156, *B. bifidum* 1005, *B. breve* 1015, and *B. breve* 1063, remained viable for 5 h after the exposure. The survival rates of the probiotic strains against lipase (700 IU/mg) were comparable to those observed with other enzymes, however, the differences were not statistically significant. All strains survived up to 5 h after treatment with lysozyme, proteinase, α-amylase, and lipase (Figure 1).

### 3.2 Survival rate of probiotics in saliva and potential for binding to teeth

Although the number of probiotic strains exposed to the saliva and teeth after 24 h was significantly decreased compared to the control group, as shown in Table 4, probiotics displayed tolerance to saliva and showed weak adherence to the teeth (*p* > 0.05).

**Table 4:** Log 10 CFU/mL of probiotics after 24 h treatment on maxillary molars and exposure to saliva.

Probiotic strains	Control	Maxillary molars	Saliva
*L. plantarum* 42	250	25	57
*L. rhamnosus* 195	200	14	30
*L. brevis* 205	150	18	40
*L. plantarum* 156	150	22	60
*L. reuteri* 100	100	31	69
*B. bifidum* 1001	250	13	35
*B. bifidum* 1005	100	29	71
*B. breve* 1015	150	13	75
*B. breve* 1063	120	15	32
*B. longum* 1044	100	14	70

### 3.3 Biofilm formation ability of probiotics

All probiotics were able to form biofilm. In Table 5, the probiotic isolates are classified based on biofilm production ability as strong, medium, weak, and non-biofilm producers. None of the probiotics showed strong abilities in producing biofilms, the maximum and minimum levels of biofilm production by probiotics were measured at 0.144 and 0.112, respectively. There was no significant difference among probiotics in biofilm production (*p* > 0.05).

**Table 5:** Biofilm formation ability of probiotics.

Probiotic strains	Biofilm formation
*L. plantarum* 42	Weak
*L. rhamnosus* 195	Weak
*L. brevis* 205	Weak
*L. plantarum* 156	Weak
*L. reuteri* 100	Weak
*B. bifidum* 1001	Weak
*B. bifidum* 1005	Medium
*B. breve* 1015	Weak
*B. breve* 1063	Weak
*B. longum* 1044	Weak

### 3.4 Aggregation and co-aggregation ability of probiotics

All probiotics showed moderate to weak auto-aggregation ability (Table 6); however, no significant difference was observed in aggregation formation between probiotics (*p* > 0.05). The present study examined the co-aggregation of probiotics with oral pathogens, and measured the reduction in optical absorbance of bacterial tubes after 8 h. The findings revealed that all probiotics were able to co-aggregate with *S. mutans*, *P. gingivalis*, *F. nucleatum*, and *A. actinomycetemcomitans*. Strong co-aggregation was observed between *B. bifidum* 1001, *L. plantarum* 156, *L. reuteri* 100, and *L. brevis* 205 with *S. mutans*; *B. bifidum* 1005 and *L. reuteri* 100 with *P. gingivalis; L. plantarum* 156 and *L. plantarum* 42 with *F. nucleatum;* and *B. longum* 1044 and *L. plantarum* 156 with *A. actinomycetemcomitans* (Table 6).

**Table 6:** Aggregation and co-aggregation ability of probiotics after 8 h incubation.

Probiotic strains	Aggregation value	Co-aggregation (%)			
		*S. mutans*	*P. gingivalis*	*F. nucleatum*	*A. actinomycetemcomitans*
*L. plantarum* 42	+	15.59	22.7	30.21	16.5
*L. rhamnosus* 195	++	25	5.12	15	17.25
*L. brevis* 205	+	45.1	7.15	18.55	15.53
*L. plantarum* 156	+	51.2	8.35	35.6	18
*L. reuteri* 100	+	49.5	46.1	15.1	15.1
*B. bifidum* 1001	++	65.57	20.38	29	5
*B. bifidum* 1005	++	15.3	50.06	16.95	4.69
*B. breve* 1015	+	24.35	21.5	17.22	7.5
*B. breve* 1063	++	15.02	20.25	17.65	15.31
*B. longum* 1044	+	15.41	8	29.5	18.7

### 3.5 Cell culture analysis

#### 3.5.1 The MTT assay

**Figure 2:**
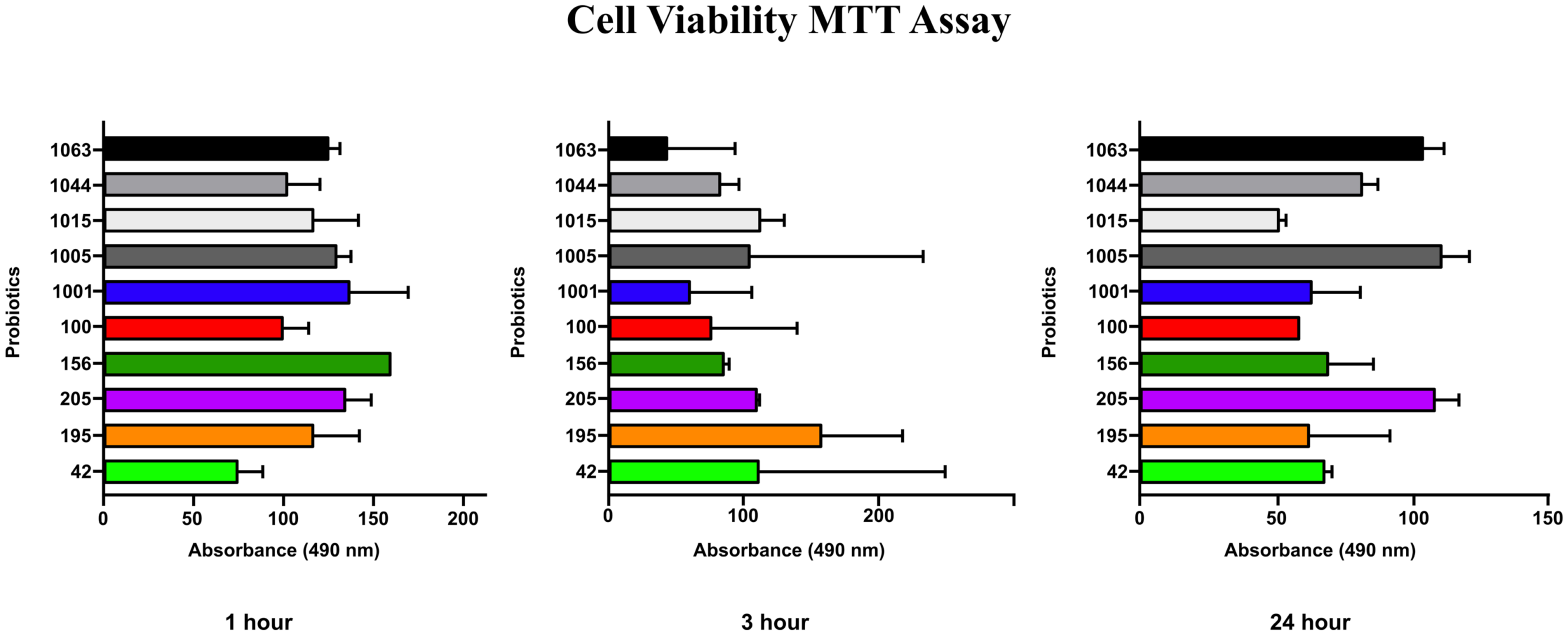
MTT assay (cell viability %) of probiotics after 1, 3 and 24 h for HGF cell line. No significant influence on gingival fibroblast cell viability was observed.

The MTT viability assay results showed that none of the probiotics had toxicity on HGFC when administered at a concentration of 10^8^ CFU/mL for 1, 3, and 24 h (Figure 2). This concentration was used in subsequent cell-related experiments. Notably, the negative control in this experiment was considered HGFC without treatment by any probiotics.

#### 3.5.2 Adhesion assays of probiotics to HGFC

*In vitro* binding of probiotics to HGFC was determined after 2 h of incubation. The findings showed that all probiotics possessed binding ability to HGFC (Table 7). As indicated in Table 7, the comparison of probiotics showed that *L. reuteri* 100 and *B. bifidum* 1,005 exhibited the highest cell attachment capability, with an average of 23.8 and 20.5 adherent bacteria per HGF cell, respectively. The present study results indicated that eight strains were found to be adhesive, while the remaining strains did not show adhesive properties.

**Table 7:** Adhesion rate of probiotic to HGFc (mean of colony count, CFU/mL).

Probiotics strains	1005	1015	1044	1063	42	205	100	195	156	1001
Adhesion rate	20.05	16.9	13.5	4.75	11.65	2.6	23.8	8.2	14.8	18.95
High adhesiveness (>40)										
Adhesiveness (6–40)	^*^	^*^	^*^		^*^		^*^	^*^	^*^	^*^
No adhesiveness (<5)				^*^		^*^				

*: Adhesiveness profile.

#### 3.5.3 Effects of probiotics on the virulence gene expression of oral pathogens by qRT-PCR

**Figure 3:**
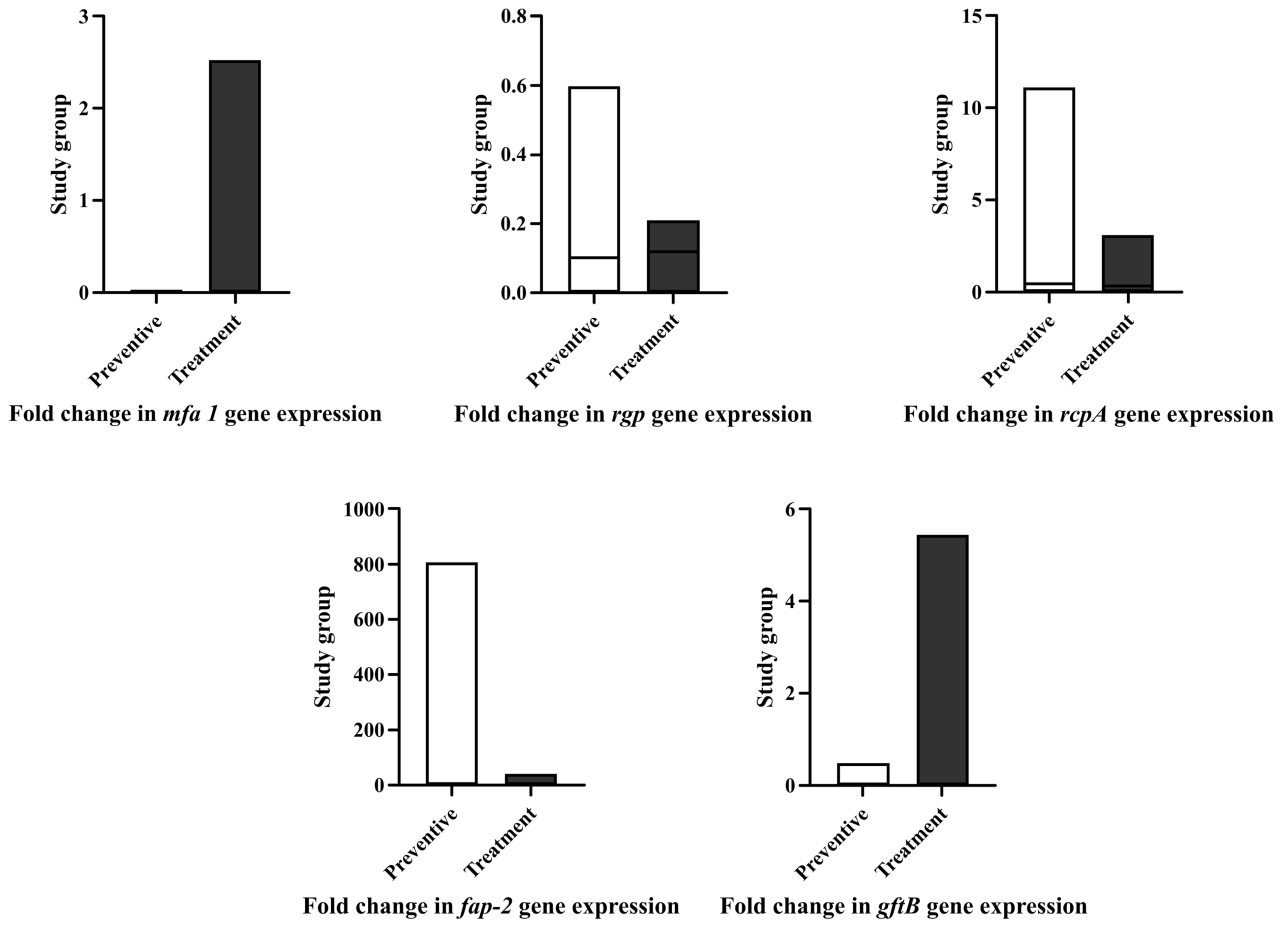
Most of single and live probiotics as well as cocktails of *Bifidobacterium* plus *Lactobacillus* in preventive and treatment group reduced the fold change of virulence gene expression in *P. gingivalis*, *A. actinomycetemcomitans*, *F. nucleatum*, and *S. mutans*. None of gene expression changes were statistically significant. All data represent as mean ± SEM.

The qRT-PCR was used to investigate the effect of probiotics on the expression of virulence genes of oral pathogens (Figure 3). Most of the living probiotics and cocktail of *Bifidobacterium* plus *Lactobacillus* in the present study changed the expression of *Mfa1* and *RgpA*, which was not statistically significant (*p* > 0.05). *L. plantarum* 42 and *B. longum* 1044 in the prevention group, *B. breve* 1015 and a cocktail of *Lactobacillus* in the treatment group, and a cocktail of *Bifidobacterium* plus *Lactobacillus* in both groups were effective in reducing *RcpA* in *A. actinomycetemcomitans* (*p* > 0.05). *L. brevis* 205, *B. longum* 1044, *B. breve* 1015, and a cocktail of *Bifidobacterium* plus *Lactobacillus* in both groups, *L. plantarum* 156 in the treatment group, and *B. bifidum* 1001 in the prevention group were effective in reducing *GtfB* in *S. mutans* (*p* > 0.05). *L. reuteri* 100, *B. bifidum* 1001, and a cocktail of *Bifidobacterium* plus *Lactobacillus* in both groups, *B. bifidum* 1005 and *L. plantarum* 42 in the prevention group, and *L. rhamnosus* 195 in the treatment group could reduce *FapA* in *F. nucleatum* (*p* > 0.05). Using Dunnett’s multiple comparison test, no statistically significant difference was found in the virulence gene expression of oral pathogens between the prevention and treatment groups (*p* = 0.9).

#### 3.5.4 Determination of IL-8 and IL-10 changes

**Figure 4:**
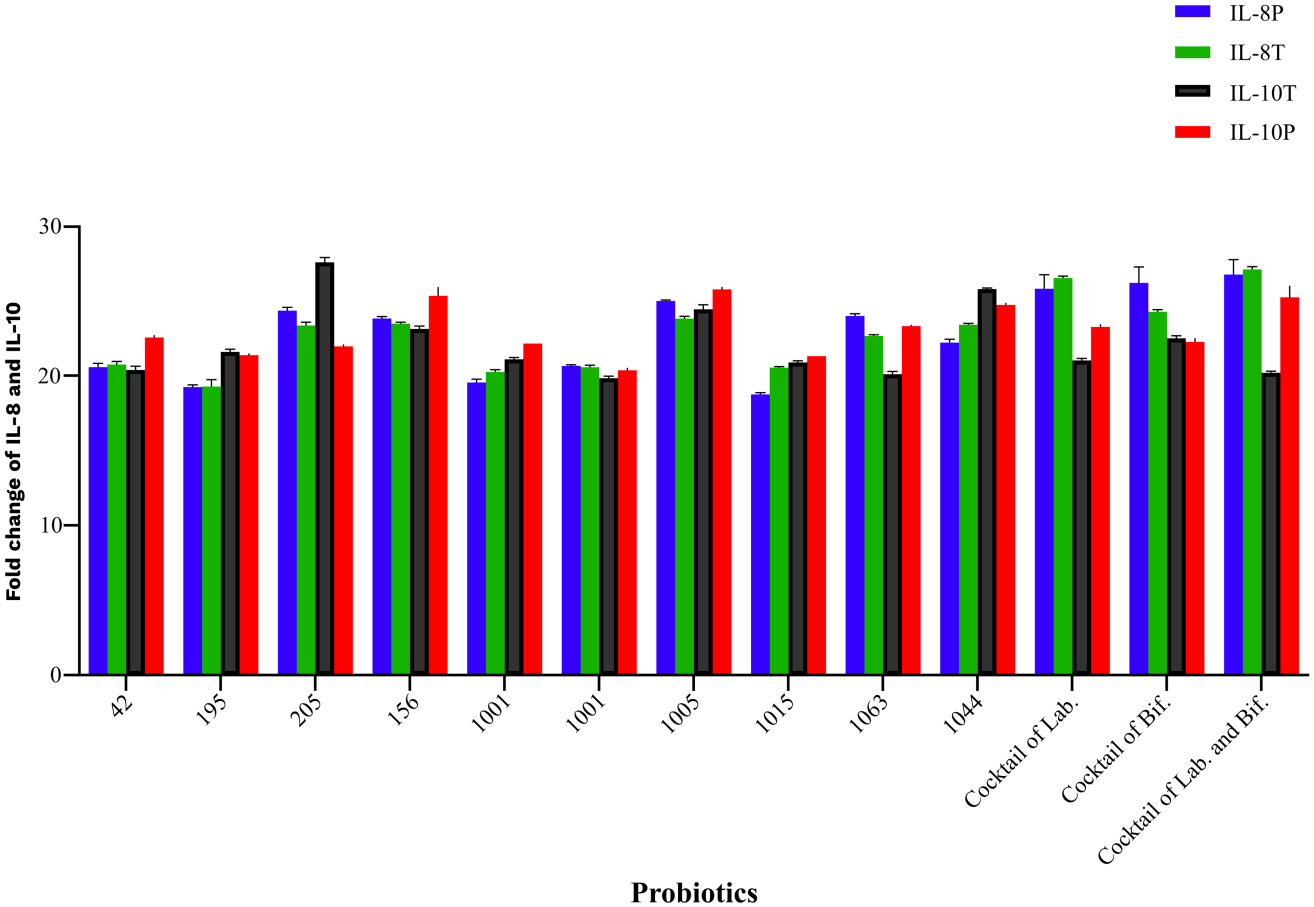
Probiotics modulate the inflammatory and non-inflammatory gene expression following challenging with *P. gingivalis* in HGF cell line. All data indicate the mean ± SEM. None of these changes were statistically significant. (P), preventive; (T), treatment; (Lab), *Lactobacillus*; (Bif), *Bifidobacterium*.

By cell culture analysis, single probiotics including *L. plantarum* 42, *B. breve* 1015, and *B. bifidum* 1001 were more effective in increasing IL-10, and *B. bifidum* 1005 was effective in decreasing 1 L-8 in both intervention groups. The present research showed that a cocktail of *Bifidobacterium* and *Lactobacillus* strains was the most effective in decreasing IL-8 and enhancing the secretion of IL-10 by HGFCs in both prevention and treatment groups. However, none of these changes were statistically significant (*p* > 0.05) (Figure 4).

### 3.6 Inhibition of biofilm formation (dental plaque) of oral pathogens by probiotics

*L. plantarum* 42 and *B. breve* 1,015 in the prevention group and *L. rhamnosus* 195, *L. plantarum* 156, and *B. longum* 1,044 in the treatment group significantly reduced the biofilm production ability of *A. actinomycetemcomitans* (*p* < 0.05), compared to the control group that did not receive any treatment. Furthermore, intra-group comparison showed no difference between the probiotics in the prevention and treatment groups in reducing *A. actinomycetemcomitans* biofilm production (*p* > 0.05). All probiotics in the prevention group were able to significantly reduce the biofilm production ability of *P. gingivalis* in comparison to the treatment group (*p = 0.01*). However, *L. reuteri* 100, *B. bifidum* 1001, *B. bifidum* 1005, *B. breve* 1015, and *B. breve* 1063 in the treatment group were able to reduce *P. gingivalis* biofilm formation, but it was not significant (*p* > 0.05). When comparing the effects of probiotics on *F. nucleatum* biofilm formation, it was observed that *L. plantarum* 42, *L. reuteri* 100, *B. bifidum* 1001, and *B. longum* 1044 in the prevention group, as well as *L. plantarum* 42, *L. brevis* 205, *L. plantarum* 156, *B. bifidum* 1001, *B. bifidum* 1005, *B. breve* 1015, and *B. longum* 1044 in the treatment group, reduced *F. nucleatum* biofilm formation in compare to the control group (*p* > 0.05).

**Figure 5:**
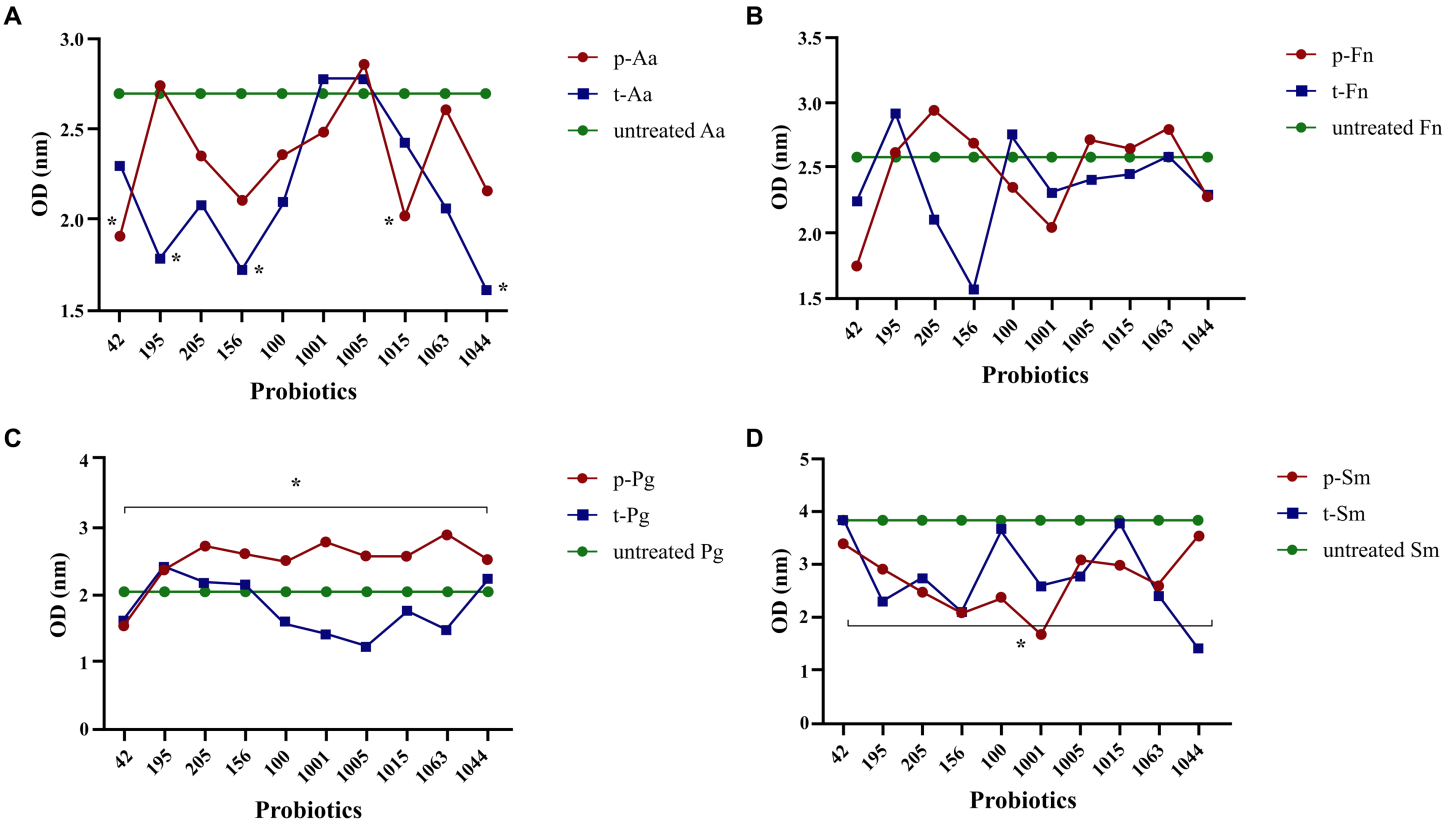
Probiotics reduced biofilm formation by oral pathogens, including *A. actinomycetemcomitans* **(A)**, *F. nucleatum* **(B)**, *P. gingivalis* **(C)**, and *S. mutans* **(D)**. All data indicate the mean ± SEM. ^*^p < 0.05. Statistically significant decrease in the biofilm production in *A. actinomycetemcomitans* was observed in both treatment and preventive groups. In all preventive group, probiotics dereased the biofilm formation in *S. mutans* and *P. gingivalis*, while the reduction was not significant for the treatment group. Biofilm formation reduction for *F. nucleatum* was not significant in preventive and treatment groups.

The group of *S. mutans* that received probiotics for prevention showed a significant decrease in biofilm production compared to the control group (*p* = 0.04). In contrast, the difference in biofilm formation was not significant between the treated and untreated *S. mutans* groups (*p* = 0.05). No significant difference was observed following an intra-group comparison between either *S. mutans* isolates receiving probiotics as a prevention or treatment group (*p* > 0.05). Notably, *L. plantarum* 156 and *B. bifidum* 1001 in the prevention group and *L. plantarum* 42 and *B. longum* 1044 in the treatment group resulted in the highest reduction in *S. mutans* biofilm formation. All results are shown in Figure 5.

## 4 Discussion

Antibiotics and mechanical removal of dental plaque are commonly employed to reduce the biofilm and periodontal pockets in dental plaque-related diseases. There is concern regarding the diminishing effectiveness of antibiotics due to the emergence of antibiotic resistance and potential side effects (Homayouni Rad et al., 2023), however, antibiotics are still used in treatment. Herbal-based medicines with antimicrobial properties like *Aloe barbadensis* Miller, *Trifolium pratense*, and *Medicago sativa* (Palombo, 2011), as well as enzyme inhibitors like protease inhibitors against *P. gingivalis* (Hosn et al., 2015), and microbial therapy like probiotics (Saraf et al., 2010), are innovative therapeutic strategies which have been developed to combat oral diseases. Medicinal plants are known for their ability to provide antioxidants and anti-inflammatory benefits, with minimal adverse effects and the rarely emergence of resistance strains (Pasupuleti et al., 2023). Designing inhibitors targeting cysteine proteases of *P. gingivalis* would be beneficial in the management of periodontitis by preventing tissue destruction (Hosn et al., 2015).

The interest in utilizing probiotics in dentistry has increased. As probiotics target the pathogens while maintaining a balance within the oral microbiota, antibiotics disrupt normal flora besides eliminating pathogenic bacteria. According to *in vitro* studies, probiotics prevent the growth of cariogenic bacteria like *S. mutans* and periodontal pathogens such as *P. gingivalis*, *A. actinomycetemcomitans*, *Prevotella intermedia*, and *F. nucleatum* by colonizing the oral cavity, forming biofilm, and reducing the acidity level, which helps to maintain hemostasis and manage the immune system (Haukioja, 2010; Homayouni Rad et al., 2023; Shirbhate et al., 2023). Furthermore, clinical trials have shown a significant decrease in probing pocket depth (PPD), clinical attachment loss (CAL), bleeding on probing (BOP), plaque index (PI), and gingival index (GI) in the probiotic group (Dhaliwal et al., 2017; Costacurta et al., 2018; Kumar et al., 2021; Hardan et al., 2022). An ideal probiotic for controlling oral diseases should effectively colonize oral surfaces, be resistant to oral flora, have the ability to form biofilm, not produce foul-smelling compounds, lack resistance to antibiotics and toxicity, establish homeostasis in the oral cavity and regulate immune responses, and not promote caries (Bosch et al., 2012; Wen et al., 2022).

### 4.1 Functional properties of probiotics in the oral cavity

The main finding of this study is the durability of probiotics isolated from breast milk and infant feces in exposure to various enzymes as well as their binding ability to gingival fibroblast cells. To determine the most resistant probiotic in the oral cavity, the concentrations of protease, lipase, and α-amylase enzymes, tested in this study were adjusted near their natural concentrations in saliva, and the results showed all probiotics were stable in the oral cavity and saliva. Probiotics that possess the capability to produce biofilm, can establish long-term colonization in the gut, effectively limiting the growth of harmful bacteria. All probiotics demonstrated weak biofilm formation and aggregation. The development of biofilm and aggregation by probiotics is primarily influenced by their ability to adhere to surfaces, and all probiotics examined in this study showed the ability to attach to maxillary molars and gingival fibroblast cells (D'Alessandro et al., 2021). Nevertheless, probiotics showed weak adhesion capabilities, *Lactobacillus* spp. demonstrated great adhesiveness compared to other probiotic strains. This supports the idea that probiotics originated from fecal samples are the same as to those found in the mouth (Rohani et al., 2015). In order to establish long-term colonization of probiotics in the oral cavity, a strong and permanent adherence is required to colonization, however, probiotics remain for a short period in the oral cavity, and most of them establish a reversible adherence with cells that can be easily detached (Van Holm et al., 2023).

### 4.2 Biofilm inhibition by probiotics

Biofilm serves as a niche for protecting microorganisms (Jayathilake et al., 2017). This study revealed, *S. mutans* exhibited a robust ability to form biofilms, although *P. gingivalis* showed weaker biofilm formation capability. Targeting biofilm formation in biofilm-producing pathogens is the most important objective in the management of caries and periodontal disease. An efficient probiotic hinders the growth of pathogens in dental plaque through biofilm formation and co-aggregation with pathogens. Our probiotics reduced the biofilm formation of *A. actinomycetemcomitans*, *P. gingivalis*, *F. nucleatum*, and *S. mutans* in the prevention and treatment groups. A significant decrease in biofilm formation of *S. mutans* was observed, which was confirmed by a significant reduction in the *GtfB* transcript. This is in accordance with observations made by Ahmed et al. (2014) and Hasan et al. (2015) that demonstrated the reduction in Gft enzyme expression plays an important role in preventing biofilm formation and developing caries in *L. rhamnosus*. These claims highlight the potential of *Lactobacillus* spp. as a promising alternative to antibiotics due to their anti-biofilm activity (Ahmed et al., 2014), and suppress the expression of *GtfB*, *GtfC*, and *GtfD* in *S. mutans* (Hasan et al., 2015). Although the main mechanism of biofilm inhibition by probiotics is still not fully understood, competing for nutrients, disrupting the attachment of pathogens, and producing antimicrobial peptides have been described as potential mechanisms (Barzegari et al., 2020). Some probiotics, like *L. plantarum*, reduce the biofilm mass by positively-charged D-Alanine in lipoteichoic acid (LTA). LTA has an inhibitory effect on the production of extracellular polymeric substances (EPS) (Ahn et al., 2018). The cationic amphiphilic structure of LTA also prevents aggregation by binding biofilm-associated genes to bacterial DNA (Anunthawan et al., 2015). In addition to LTA, probiotics such as *L. fermentum* can limit the biofilm production in *S. mutans* by using antibiofilm substances like biosurfactants (Tahmourespour et al., 2011). It is similar to the suppression of biofilm formation in *Actinomyces naeslundii* and *Staphylococcus aureus* through rhamnolipid and lipopeptide production by *Burkholderia thailandensis* and *Bacillus subtilis*, respectively (Rivardo et al., 2009; Elshikh et al., 2017). Interestingly, probiotics prevent the production of biofilm by other pathogenic bacteria due to their biofilm formation ability. According to the Ramos et al. study (Ramos et al., 2012), the supernatant of *L. plantarum* reduced the formation of *P. aeruginosa* biofilm without changing its matrix composition. By viability assay, they showed lactic acid solution prepared from *L. plantarum* not only has bacteriostatic and bactericidal properties on biofilm and planktonic forms of *P. aeruginosa*, but also suppresses the expression of virulence factors which are regulated by quorum sensing (QS), like elastase, rhamnolipid, and pyocyanin (Durant et al., 2000; Ramos et al., 2012). Differences in bacterial source, LTA structure, and probiotic strain led to controversies in biofilm inhibition results (Ryu et al., 2008).

### 4.3 Antimicrobial and anti-inflammatory effects of probiotics on oral pathogens

Studies have shown that probiotics exert antimicrobial properties through the production of enzymes, acids, bacteriocins, and hydrogen peroxide (Duncker et al., 2011). In addition to preventing biofilm formation, the present study also showed *L. plantarum* 42, *L. reuteri* 100, and *B. bifidum* 1001 have inhibitory impacts on gene transcripts of *RcpA*, *Mfa1*, *RgpA*, *Fap2*, and *GtfB* in treated *A. actinomycetemcomitans*, *P. gingivalis*, *F. nucleatum*, and *S. mutans* compared to the untreated control group and other probiotics. The aforementioned genes are involved in biofilm formation (Smith et al., 2016), colonization, auto-aggregation, and the interaction of pathogens with the surrounding (Zijnge et al., 2012).

Probiotics also regulate host-related factors and modulate inflammation in the oral cavity. In this study, probiotics were effective in decreasing IL-8 and increasing IL-10 in HGFCs in both prevention and treatment groups. This is similar to the Kaci et al. (2011, 2014) findings which showed *S. salivarius* reduced TNF-α, IL-1, and IL-8 in stimulated intestinal epithelial cells and HT-29 by a small heat-resistant protein produced by some probiotics. Probiotics also modulate inflammation by producing short-chain fatty acids (SCFA) as postbiotic compounds like propionate and butyrate. Postbiotics produced by probiotics not only suppress the NF-κB pathway and interfere with suppression of Treg function (Vinolo et al., 2011), but also stimulate the production of anti-inflammatory cytokines and Treg differentiation (Kespohl et al., 2017).

The efficacy of our probiotics did not meet significant results as expected, which was similar to the Bosch et al. (2012) study. Lack of significant impressiveness by probiotics arises from their limited potential in biofilm formation, aggregation, and adhesion to HGFCs, which determine the eligibility of a candidate probiotic for use in the treatment of oral diseases (Sorroche et al., 2012). Moreover, the pathogen-specific inhibitory effect of probiotics and the presence of thick peptidoglycan in Gram-positive bacteria (Yang et al., 2021), should be considered which require a higher dosage of probiotics and a cell-free culture supernatant to significantly prevent the growth of harmful bacteria (Ding et al., 2021). In this context, Yang et al. (2021) showed that cell-free culture supernatant of *L. reuteri* had greater antimicrobial activity than live strains, due to the production of secondary bioactive metabolites (Ren et al., 2018), and the reduction of intracellular ATP required for the growth of oral pathogens. This evidences encourages us to prioritize the use of cell-free culture supernatants in future studies. In the present study, *L. reuteri* represents great function in tolerating oral conditions, binding to gingival fibroblast cells, and reducing biofilm formation, virulence gene expression, and inflammatory responses. The beneficial effect of *L. reuteri* and its survival in rough conditions were already proven and attributed to the production of inulin-type fructansucrase (Duncker et al., 2011), reutericyclin, reuteran, and reuterin (Sun et al., 2022).

### 4.4 Oral probiotics vs. fecal probiotics in the management of oral disease

To determine whether the origin of the probiotic isolation involved in lack of significant results or not, the functional equivalences and differences of oral and fecal probiotics should be addressed. Shimabukuro et al., in an animal model, evaluated the management of periodontitis by *B. breve* and *B. bifidum*, which were extracted from fecal samples. They showed complete clearance of *P. gingivalis* from oral biofilms and improvement in alveolar bone destruction by *B. bifidum* (Shimabukuro et al., 2021). The study evaluated the adherence ability and the modulatory effect of the immune system of *L. salivarius* AR809 extracted from the oral cavity of a healthy individual (Jia et al., 2019). The efficient adherence of oral isolated probiotics to pharyngeal epithelial FaDu cells and modulating the host’s immune response by enhancing IL-10 production and reducing the expression of TNF-α and IL-1B were observed. Despite being less studied about the effect of probiotics on dental caries disease and the need for further comprehensive studies, recent studies have shown probiotics isolated from oral and fecal sources to be promising candidates for clinical application (Shokryazdan et al., 2017).

### 4.5 Probiotic administration strategies and challenges

The main challenges in the widespread application of probiotics for oral treatment are the introduction of single probiotic strains and the limited global access to probiotics (Spacova et al., 2020). Another important consideration in probiotic use is patient acceptance. However, following the use of mouthwash-containing probiotics, patient compliance was evaluated which more than 95% of volunteers were satisfied (Nisha et al., 2023). The effectiveness of probiotics also depends on the delivery method in terms of fluctuations of temperature, oxidation, pH level, and resistance to decomposition by enzymes. Various methods have been introduced to overcome delivery limitations, among them oral delivery is more common due to patient compliance, cost-effectiveness, and easy application for prevention and treatment purposes (Baral et al., 2021). Oral delivery of probiotics is achieved by dietary supplements, oral suspensions, lozenges, mouthwash, granules, capsules, oral films, and tablets for the treatment of oral diseases (Lee et al., 2017; Nie et al., 2023). Mouthwash-containing probiotics contribute to reducing plaque formation and compete with pathogens for adhesion sites, thereby regulating plaque ecology in normal flora (Meurman and Stamatova, 2007). Chewing gums are another common formulation of probiotics for oral diseases which are designed by probiotics (Ribeiro et al., 2020). Probiotic based gums successfully prevent *S. mutans* growth, and manage periodontal diseases (Krasse et al., 2006). The simple way to administer of probiotics is through dietary supplements in the form of granules to achieve a gradual and slow release of probiotics (Shirbhate et al., 2023).

### 4.6 The safety concerns of probiotics

Despite the safety history of probiotics for clinical application, a rarely occurring bacteremia in high-risk individuals was reported following consumption of probiotics (Doron and Snydman, 2015). Therefore, it is essential to properly examine the characteristics of microbial therapy with probiotics. The general safety of probiotics is described as their stability against rough conditions like gastric juice and enzymes, and their the binding ability, while functional safety is determined by having an antagonistic ability against pathogens, modulating immune responses, and selective activity in stimulating or suppressing the growth of certain bacteria in the oral cavity (Shirbhate et al., 2023). In addition to the survival of probiotics in the oral cavity, we have previously confirmed that present probiotics are resistant at pH = 2 and 0.4% bile salt, do not harbor any antibiotic resistance genes, or induce hemolysis (Rohani et al., 2015; Eshaghi et al., 2017). Also, the safety of present probiotics based on their mutagenicity, genotoxicity, and possible adverse effects was shown in the Darbandi et al. (2022) study, in which no mutations, genotoxicity in cell line examination, or adverse effects in animal models like mortality, abnormality, and weight change were observed in any dose range of 2000, 1,000, and 500 mg/kg. The examination of probiotics to produce lactic acid is important. Bosch et al. (2012) excluded 7 out of 46 probiotic candidates due to lactic acid production and the risk of dental caries.

The long-term effect of probiotics in the oral cavity is not well-known, but the short-term effects of probiotics showed they have significant anti-caries impacts, and significant changes in the alpha diversity of the oral microbiome (Dassi et al., 2018). Hradicka et al. (2023) studied the safety concerns of long-term probiotics in people who have taken probiotics daily for more than 10 years. They showed the long-term administration of probiotics does not have a significant effect on health, and causes gut microbiota alteration, and a significant increase in serum biochemical parameters, lipid metabolism, and inflammatory response. Meanwhile, the short-term administration of probiotics has significant effects on strengthening the gut-liver axis pathway and stimulating the innate immune response. This claim emphasizes the importance of the duration of probiotic consumption in the outcome of the clinical application.

### 4.7 Future prospects and suggestions

For future prospective research, evaluation of the safety of probiotics, the composition of the microbiota before and after the administration of probiotics, the optimal dosage, the duration of administration, and determining whether preventive or treatment purposes were recommended to be considered. Despite the various animal model studies that have been conducted to investigate caries-related disease, rats are not an ideal representation of humans. Therefore, probiotics should be studied in all human groups, especially high-risk populations such as immunocompromised individuals and patients with severe underlying diseases. Also, it is important to consider the characteristics of the participants based on gender and lifestyle habits (smoking, drugs, alcohol, and lifestyle) in a long-term clinical trial.

### 4.8 Limitations and strengthening

The current study did not investigate the production of lactic acid, unpleasant volatile compounds, hydrogen sulfide, and soluble glucan by probiotics, as well as the scanning electron microscope (SEM) analysis that could provide more insight into microbial communities. These experiments will be examined in upcoming studies. In this study, different combinations of probiotics in cocktails were used to determine their efficacy against oral pathogens. The key distinction of the current study from similar studies lies in the source of probiotics utilized. While many studies have focused on the effects of commercially available oral probiotic tablets or isolated strains, the present study employed probiotics derived from feces and breast milk, which has shed light on their potential for oral treatment. Further studies are needed to examine their protective role in the mouth and confidently introduce all or some of them as potential candidates for the treatment of dental caries disease.

## 5 Conclusion

According to the specific criteria of selection for oral application of probiotics, the functional capabilities of present probiotics showed they had the ability to survive in the oral cavity, form biofilm and aggregation with oral pathogens, modulate immune responses, prevent the growth of pathogens, and adhere to the HGFC and maxillary molar. However, some of these effects were not statistically significant. Moreover, no antibiotic resistance, mutagenicity, toxicity, or genotoxicity were reported from the aforementioned probiotics. Our results also confirm the direct relationship between biofilm production, aggregation and co-agglutination ability, which significantly depends on the adherence ability of bacteria. Our finding discovered *L. reuteri* as a promising probiotic candidate which effectively reduces the expression of virulence genes, inhibits biofilm formation, remains stable in oral cavity conditions, and binds to gingival fibroblast cells. For future study, we suggest using the cell-free culture supernatant of *L. reuteri* to treat or prevent caries and periodontitis.

## Data availability statement

The original contributions presented in the study are included in the article/supplementary material, further inquiries can be directed to the corresponding authors.

## Ethics statement

The studies involving humans were approved by Ethics Committee of the Iran University of Medical Sciences (IR.IUMS.FMD.REC.1400.544). The studies were conducted in accordance with the local legislation and institutional requirements. The participants provided their written informed consent to participate in this study.

## Author contributions

MM: Conceptualization, Data curation, Formal analysis, Investigation, Methodology, Project administration, Resources, Software, Validation, Visualization, Writing – original draft, Writing – review & editing. SM: Formal analysis, Writing – review & editing. DD-S: Data curation, Writing – review & editing. AD: Formal analysis, Writing – review & editing. SR: Supervision, Writing – review & editing. MT: Conceptualization, Data curation, Formal analysis, Funding acquisition, Investigation, Methodology, Project administration, Resources, Software, Supervision, Validation, Visualization, Writing – review & editing.
